# Community Engagement in Health-Related Research: A Case Study of a Community-Linked Research Infrastructure, Jefferson County, Arkansas, 2011–2013

**DOI:** 10.5888/pcd12.140564

**Published:** 2015-07-23

**Authors:** M. Kathryn Stewart, Holly C. Felix, Mary Olson, Naomi Cottoms, Ashley Bachelder, Johnny Smith, Tanesha Ford, Leah C. Dawson, Paul G. Greene

**Affiliations:** Author Affiliations: Holly C. Felix, Ashley Bachelder, Leah C. Dawson, Paul G. Greene, Fay W. Boozman College of Public Health, University of Arkansas for Medical Sciences, Little Rock, Arkansas; Mary Olson, Naomi Cottoms, Tri County Rural Health Network, Inc, Helena, Arkansas; Johnny Smith, Shiloh Baptist Church and Ten Thousand Black Men, Pine Bluff, Arkansas; Tanesha Ford, University of Arkansas at Pine Bluff, Pine Bluff, Arkansas.

## Abstract

**Background:**

Underrepresentation of racial minorities in research contributes to health inequities. Important factors contributing to low levels of research participation include limited access to health care and research opportunities, lack of perceived relevance, power differences, participant burden, and absence of trust. We describe an enhanced model of community engagement in which we developed a community-linked research infrastructure to involve minorities in research both as participants and as partners engaged in issue selection, study design, and implementation.

**Community Context:**

We implemented this effort in Jefferson County, Arkansas, which has a predominantly black population, bears a disproportionate burden of chronic disease, and has death rates above state and national averages.

**Methods:**

Building on existing community–academic partnerships, we engaged new partners and adapted a successful community health worker model to connect community residents to services and relevant research. We formed a community advisory board, a research collaborative, a health registry, and a resource directory.

**Outcome:**

Newly formed community–academic partnerships resulted in many joint grant submissions and new projects. Community health workers contacted 2,665 black and 913 white community residents from December 2011 through April 2013. Eighty-five percent of blacks and 88% of whites were willing to be re-contacted about research of potential interest. Implementation challenges were addressed by balancing the needs of science with community needs and priorities.

**Interpretation:**

Our experience indicates investments in community-linked research infrastructure can be fruitful and should be considered by academic health centers when assessing institutional research infrastructure needs.

## Background

Racial disparities in health and health care quality are well documented (1). An important barrier to addressing such disparities is low levels of research participation among those groups that experience disparities in illness and death rates (2). This imbalance in research creates challenges in understanding and developing strategies to address the causes of disparities (3). Multiple factors have been identified as affecting minority participation in research, including lack of trust, power differences, limited access to health care and research opportunities, participant burden, and lack of perceived relevance (2–6). Community engagement has been identified as a way to address these factors. True engagement of underrepresented communities is facilitated by intentional structural supports such as establishing community advisory boards, developing financial and other resources, involving minority researchers, hiring community health workers, sharing resources with community partners, and using community-based participatory research approaches (7–9). We describe an enhanced model of community engagement in which we developed a community-linked research infrastructure to involve minorities in research both as participants and as partners engaged in issue selection, study design, and implementation.

## Community Context

This community-linked research infrastructure (infrastructure) was implemented in Jefferson County, Arkansas, which has a predominantly black population (55.6%), bears a disproportionate burden of chronic illness, and has disease and death rates exceeding state and national averages (Table 1).

**Table 1:** Sociodemographic Characteristics and Selected Health Indicators, Jefferson County, Arkansas, Arkansas State, and United States

Characteristic or Indicator	Jefferson County	Arkansas	United States
**Sociodemographic indicators, 2009–2013** (10)			
Total population	73,191	2,958,765	318,857,056
White, %	41.8	79.9	77.7
Black, %	55.6	15.6	13.2
Bachelor’s degree or higher, %	17.5	20.1	28.8
Median household income, $	37,140	40,768	53,046
Annual average unemployment rate, %	13.4	8.9	9.7
Population under federal poverty level, %	23.9	19.2	15.4
Households with single parent, %	22.5	18.0	17.9
**Health indicators, age adjusted rates per 100,000, 1999–2013 (** 11 **)**			
Overall mortality, all causes	1,080.3	1,017.3	823.5
Cancer mortality rate among blacks	236.1	245.8	222.0
Cancer mortality rate among whites	206.4	202.0	185.2
Heart disease mortality rate among blacks	315.7	304.2	262.9
Heart disease mortality rate among whites	287.6	239.3	205.4

## Methods

The infrastructure, funded from 2010 through 2013 by the National Institute on Minority Health and Health Disparities, deployed intentional structural supports identified in the literature as facilitating true community engagement (7,8). An existing long-term community–academic partnership between the Fay W. Boozman College of Public Health (the College), University of Arkansas for Medical Sciences (UAMS), and Tri-County Rural Health Network (Tri-County) developed the grant proposal to build community infrastructure for engaging minorities in research. As such, all infrastructure activities focused on this overall community engagement goal rather than on engagement in a specific program to address a particular health issue. 

Intentional structural supports designed for the infrastructure consisted of an implementation team; a community advisory board to advise on infrastructure activities; the Community Health and Research Connector Program (connector program) staffed by local community health workers, called “connectors,” to address cultural, social, and community factors affecting access to and use of health-related resources (eg, social services, medical care, research opportunities); a health registry to provide the community advisory board with input from a large segment of the community regarding health needs and use of health-related resources; a resource directory to facilitate community access to health-related resources; and a research collaborative to solicit ideas on research priorities from community organizations. Tri-County conducted community forums to engage communities in dialogue about research relevant to health disparities. Forums and community advisory board meetings identified concerns about disparities in chronic disease, lack of health resources, and sociocultural barriers to health care and health care use that defined the focus of the infrastructure. The Figure illustrates the contributions the implementation team made to develop and deliver the main components of the infrastructure and the associated outcomes that the components were conceptualized to affect. The UAMS institutional review board reviewed all infrastructure activities and determined that the service activities of the connector program did not constitute research.

**Figure:**
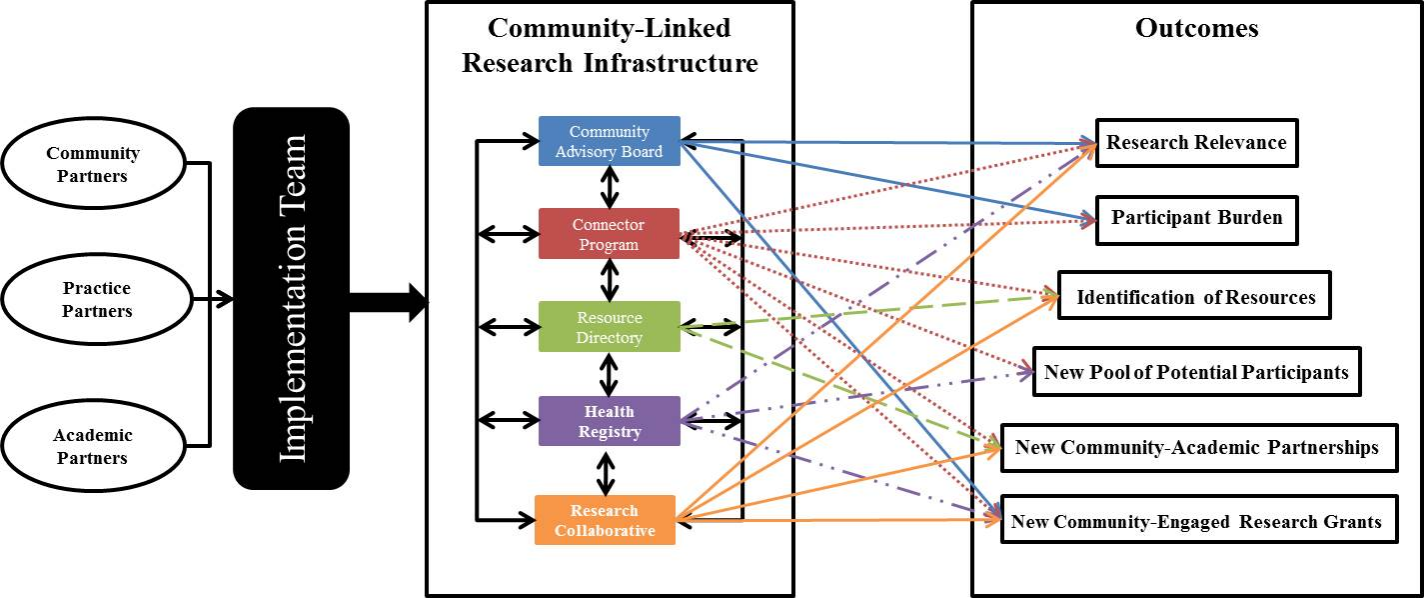
Conceptual framework of the infrastructure.

The objectives of the infrastructure were to hire and train local community health workers, contact at least 1,200 community residents, document residents’ health concerns and conditions, assess residents’ interest in health-related service programs and research projects, link residents to health-related resources, and facilitate minority community–academic research partnerships. We report on challenges addressed and short-term successes in designing and deploying this community engagement infrastructure.

### Intentional structural supports

**Implementation team.** The implementation team of grassroots, academic, and health practice community members included representatives from Tri-County, Shiloh Baptist Church, the College, and the UAMS South Central Regional Program in Jefferson County (South Central) (Table 2). In addition to managing overall efforts, the team facilitated community-partnered research by introducing community partners to noninfrastructure researchers with expertise related to health issues prioritized by the community and by soliciting interest from researchers with expertise in responding to community concerns.

**Table 2:** Implementation Team, Case Study of a Community-Linked Research Infrastructure, Jefferson County, Arkansas, December 2011–April 2013

Organization	Project Purpose
**Community partners**	
Tri-County Rural Health Network (Tri-County)	Nonprofit community-based organization with mission of improving access to health care. Tri-County’s primary initiative was the original Community Connector Program (12) adapted for the infrastructure. The original Community Connector Program deploys community health workers to connect people with long-term care needs to home- and community-based care by addressing socio -cultural barriers, establishing trust, and increasing access through system navigation. Medicaid now funds the original Community Connector Program based on cost savings generated through decreased use of institutional care (12). Tri-County was represented on the implementation team by its community engagement specialist. The executive director served on the community advisory board and co-chaired the research collaborative.
Shiloh Baptist Church	The pastor of Shiloh Baptist Church also directs a community-based organization in Jefferson County that supports the education, health, and employment of black men. He served on the implementation team, chaired the community advisory board, and was a member of the research collaborative.
**Academic partner**	
Fay W. Boozman College of Public Health (the College), University of Arkansas for Medical Sciences (UAMS)	Faculty brought expertise in community-based participatory research, prevention and management of chronic illness, health disparities, research ethics, and program evaluation and the College’s long-term relationship with Tri-County Rural Health Network (13). The faculty was represented on the implementation team.
**Practice partner**	
UAMS South Central Regional Program in Pine Bluff, Jefferson County (South Central)	Local clinical resource with a community health clinic and a family practice residency program where medical, nursing, pharmacy, and allied health professionals also train. South Central was represented on the implementation team and the administrator co-chaired the research collaborative.

**Community advisory board.** The 8-member community advisory board was established to integrate the voice of the grassroots community into infrastructure design and implementation. Community partners on the implementation team invited selected community residents and representatives of community organizations with personal experience and knowledge of health and social issues in Jefferson County to serve on the community advisory board. Advisory board members received training on community-based participatory research and health disparities before being asked to sign a memorandum of understanding outlining their commitment to the infrastructure and the benefits of participation. Members were paid an honorarium to recognize their contributions in quarterly meetings.

**Community Health and Research Connector Program.** The connector program was designed as a public service provided by connectors to directly engage community residents and facilitate their access to and use of health-related resources, including both services and research opportunities. This program was adapted from another community health worker program, called the Community Connector Program, which successfully promoted use of community-based long-term care services as a cost-effective alternative to institutional care (12).

The connector program aimed to serve 1,200 minority and low-income community residents. Research participation was not required for assistance from connectors. Connectors were responsible for administering the registry questionnaire to document health needs and research interests, sustaining contact with community residents to develop trust and bi-directional interaction, facilitating connections to needed resources, providing follow-up, establishing liaisons with referral sites, entering data into an electronic database, and conducting quality control procedures to maintain data integrity.

Black residents of Jefferson County were hired as connectors. Tri-County employed the connectors rather than UAMS because employment by a community organization was seen as representing community interests and built trust with community residents. This strategy allowed us to allocate salary support for connectors to the Tri-County subcontract consistent with efforts to build financial support for community organizations and jobs to strengthen the local economy.

Community and academic partners shared responsibility for training the connectors. Tri-County assumed primary responsibility for training on civic rights and responsibilities, leadership, interpersonal communication, first impressions, understanding and processes for reaching the community, cultural and linguistic competence, safety, needs assessments, conducting forums, and developing resource directories. Researchers from the College took the lead in training on community-based participatory research and health disparities and on data collection, data entry, and quality control. Connectors also became certified in Human Subjects Protection in Research through the Office for Human Research Protections of the US Department of Health and Human Services and the Health Insurance Portability and Accountability Act (HIPAA).

Connectors engaged community members by helping them access community services: primary and specialty care, prescription assistance, and post-hospitalization follow-up; financial support for utilities, food, and housing; and assistance with re-entry into society from prison. Connectors primarily made contact with community members through regular visits to offices of health and social services, churches, community events, and through formal referral agreements with specific providers. For example, the connector program had an agreement with South Central to allow connectors to staff a desk in the clinic’s waiting room, providing community members with direct access to connector services. In this context of service, connectors collected community members’ contact and basic demographic information, invited them to complete the registry health questionnaire, and elicited community interest in research by asking community members whether they would be willing to be contacted about research opportunities.

**Health registry.** A health registry was designed to collect contact information, to monitor the health needs and interests of people served by the Connector program, to provide the community advisory board with community input regarding health status and use of health-related resources, and to monitor the connector program. Connectors collected data using both paper and electronic records. Forums and community advisory board meetings initially identified a broad scope of issues for potential inclusion in the registry health questionnaire. Academic partners selected items from validated instruments to assess these issues. Community partners reviewed proposed items and helped select those that best captured community concerns while minimizing participant burden. These items were incorporated into a draft presented to the community advisory board in a discussion of survey research methods followed by a group cognitive interviewing exercise. After input from connectors and the implementation team, the community advisory board’s feedback was incorporated into a revised version, which included 119 closed-ended items and 1 open-ended question about health concerns (Table 3).

**Table 3:** Health Registry Questionnaire, Case Study of a Community-Linked Research Infrastructure, Jefferson County, Arkansas, December 2011–April 2013

Domain	Number of Items	Source of Items
Health concerns	1 open-ended and 14 pre-coded	Community partners
Medical history	23	BRFSS (14)
Cancer screening	8	
Service use	6	
Stressors	6	
Diet, exercise, tobacco use	19	
Healthcare quality	8	
Sociodemographic characteristics	7	
Research participation	26	HINTS (15); implementation team
Permission to recontact	2	Implementation team

Abbreviation: BRFSS, Behavioral Risk Factor Surveillance System.

**Resource directory.** The resource directory developed by the connectors contained information about health and social services and research opportunities. Connectors developed relationships with the staff of organizations providing services and documented information in the directory to which they referred when linking community residents to needed services.

**Research collaborative.** We formed the research collaborative to facilitate communication, resource development, and community-engaged research. Tri-County’s executive director and South Central’s administrator co-chaired the collaborative. Other members represented the regional hospital, the local public health unit, Blue Cross Blue Shield of Arkansas, the Arkansas Department of Corrections, Shiloh Baptist Church, and the College.

## Outcome

### New community–academic research partnerships

The implementation team was successful in engaging minority community representatives as partners in research. The College’s researchers made introductions and invited noninfrastructure researchers to team meetings and community advisory board meetings in the community, and Tri-County followed up to explore collaborative opportunities. Eighteen researchers assisted by the infrastructure were sent a brief survey by email to assess their experiences. All reported positive interactions with the infrastructure. They had varying levels of engagement ranging from assistance in grant planning to implementation of community-engaged research. Researchers also reported how they had benefited from networking facilitated by the infrastructure and from Tri-County staff or the community advisory board chair and how connectors assisted in recruiting volunteers for their studies. Researchers reported that infrastructure community partners consulted with them about their ongoing or potential future community-engaged research projects, contributed to the development of funding proposals, and served as community co-investigators on funded grants. Connectors also served on numerous community advisory boards for researchers.

As of August 2014, the infrastructure facilitated submission of 23 grants involving more than 100 community partners and collaborating organizations and 46 researchers from 9 academic institutions. One example that included minority community partners and minority researchers introduced through the infrastructure focused on measuring trust. Other studies growing out of new partnerships involved the faith community’s work on mental health (16,17). Other research topics in grants submitted were substance abuse, social networks, sexual violence, pesticide exposure, and health literacy.

### Community advisory board

The community advisory board played an active role in establishing the infrastructure’s focus on facilitating research addressing health disparities, creating the registry questionnaire, and testing use of an electronic audience response system for group administration of the questionnaire to community residents. Community advisory board members reviewed aggregated registry data to assist with data interpretation, clarify needed services, identify research issues for further study, and express concerns about risks to participants or the community.

Some community advisory board members had severe health problems that sometimes affected their level of engagement. The connector program was a critical component, because connectors often helped them meet their needs. In one case, clinicians on the implementation team and community advisory board helped a member access badly needed surgical care.

### Community Health and Research Connector Program

The opportunity to link residents to services was fulfilling for those employed as connectors. However, salary support based on grant funding and an emphasis on defined procedures for data collection created challenges in retaining qualified connectors. We learned we needed to hire connectors who understood the value of research and had a strong commitment to the infrastructure. For example, one connector had had a very positive research experience herself, which enabled her to explain on a personal level why research participation is important.

The regulatory training was a challenge for some connectors who had little or no previous experience with online training. Connectors who completed the training without difficulty served as role models and tutors.

Connectors exceeded service goals by serving 2,665 black and 913 white community residents from December 2011 through April 2013. Among these residents, 85% of blacks and 88% of whites were willing to be contacted again about research that might be of interest to them.

### Health registry

Barriers to completing the health questionnaire were tensions between community concerns about survey length and wording of standardized items and researchers’ interest in covering common causes of disease and death and using validated questions for comparison purposes. These tensions were addressed by facilitating community advisory board and connector input in the design phase, incorporating community-generated questions, and allowing connectors the flexibility of collecting only contact information and ascertaining community residents’ willingness to be re-contacted with or without the shorter questionnaire sections on health concerns and medical history.

### Resource directory

Connectors were successful in developing relationships with referral agencies potentially able to provide residents with needed resources. Resources with the potential to influence interest in and access to preventive health care and research were assistance with rent and utility bills and enrollment in Medicaid and pharmacy assistance programs.

Transportation between Jefferson County and Little Rock, where more clinical studies are conducted, was a barrier to research participation, and although we engaged many investigators in community research partnerships, we were less successful in finding clinical studies focused on diseases of interest to community residents (eg, lupus).

### Research collaborative

Research collaborative members met regularly to discuss organizational issues and concerns related to health disparities. For example, representatives from the Arkansas Department of Corrections described high rates of pregnancy among newly released female inmates, which led to collaborative efforts between the Arkansas Department of Corrections, the Jefferson County health unit of the Arkansas Department of Health, the South Central Clinic, and the connectors to facilitate access to family planning services in the community before release. In addition, regional hospital representatives discussed concerns about avoidable hospital readmissions and the possibility of a study to test use of connectors to help reduce readmissions. Toward that aim, connectors and hospital administrators established HIPAA-compliant procedures to refer patients to connectors as a routine component of hospital discharge to facilitate connector assistance.

## Interpretation

This article describes a community engagement effort to increase involvement of minorities as research partners and to identify minorities in the community interested in learning about opportunities to participate in research. By implementing intentional structural supports (ie, community–academic partnerships, community advisory board, community health worker model, health registry, resource directory, research collaborative), we were able to engage the broader community in research and successfully reach populations with disproportionate health burdens. UAMS investigators, including black, white, and other minority researchers, have engaged with community organizations, and black community residents now serve as community co-investigators on new studies focused on issues of importance to the community (16,17). The adaptation of the Community Connector Program (12), the integration of institutional providers’ assistance with referrals and connections, and the community engagement approach of the infrastructure created a context in which access was increased and minorities were engaged who often are not represented in research.

Our work is instructive in the context of the efforts of others to increase minority research engagement. Chadiha et al have also reported the use of community-based participatory research, including a community advisory board, to build a research volunteer registry of senior urban blacks (18). They were able to enroll 1,273 volunteers in the registry over 7 years by recruiting at events sponsored by the health research center. At least 9 researchers successfully used their registry to recruit study participants over 5 years. In comparison, the infrastructure was able to engage a larger population of potential research participants in a shorter period, perhaps because it was more community-driven, hired local connectors through a community organization instead of relying on volunteers, and targeted both community organizations and individual residents for engagement.

The connectors were able to engage and provide information to a predominantly minority population often underrepresented in research, which encouraged the population’s interest in learning about research opportunities. Eighty-five percent of blacks and 88% of whites were willing to be contacted again about research that might be of interest to them. This finding is consistent with the review conducted by Wendler et al of 20 studies reporting consent rates by race or ethnicity. That review found few differences between blacks and whites in their willingness to participate in research and suggested that access is a greater barrier than attitudes (6).

A survey of community health worker programs in 2011 found that three-fourths of respondents were involving community health workers in research activities; 39% of respondents reported involvement of community health workers in research recruitment (19). A more recent multisite study conducted by Cottler and colleagues at 7 Clinical and Translational Science Award (CTSA) Sentinel Network sites found blacks were more willing than other racial groups to participate in research, even when it required obtaining blood samples (20). That study, like the infrastructure, hired community health workers to engage residents directly, and the authors concluded that the workers were crucial to research success. The community health workers were hired by the institutional members of the CTSA Sentinel Network and focused on direct engagement of potential participants rather than building partnerships with the organizations by which they are served to be sure they did not “bypass the input of community members or inadvertently privilege the perceptions of community leaders and service providers” (20). Although this approach may be effective in recruiting people for clinical trials and avoiding censoring and interpretation of community residents’ research interests by organizational gatekeepers, engagement at both individual and organizational levels allowed the infrastructure to facilitate development of new community–academic partnered studies and programs and engage minority investigators in community-engaged research while also identifying individual-level interest in research.

Although both the infrastructure and the Sentinel Network successfully engaged minorities in research through the work of community health workers, sustainability of interventions employing community health workers has traditionally been a challenge. Research infrastructure funding, such as CTSA resources or research center grants, is potentially an ideal source of support for community health workers. Although the grant period that supported the development of the infrastructure is complete, Tri-County managed to continue support for some of their connectors through grants on which they are partners. New organizational partnerships facilitated through the infrastructure will likely continue well into the future. We believe our experience with the infrastructure indicates that investments in community-based research infrastructure should be considered by academic health centers when assessing institutional research infrastructure needs.
